# Functional Assessment of Anal Sphincter with Transperineal Ultrasound and Its Relationship to Anal Continence

**DOI:** 10.3390/diagnostics14232614

**Published:** 2024-11-21

**Authors:** Yaman Degirmenci, Joscha Steetskamp, Roxana Schwab, Annette Hasenburg, Markus Schepers, Ina Shehaj, Christine Skala

**Affiliations:** 1 Department of Gynecology and Obstetrics, University Medical Center of Johannes Gutenberg University, 55131 Mainz, Germany; joscha.steetskamp@unimedizin-mainz.de (J.S.); roxana.schwab@unimedizin-mainz.de (R.S.); annette.hasenburg@unimedizin-mainz.de (A.H.); ina.shehaj@unimedizin-mainz.de (I.S.); christine.skala@unimedizin-mainz.de (C.S.); 2 Institute of Medical Biostatistics, Epidemiology, and Informatics (IMBEI), University Medical Center of Johannes Gutenberg University, 55131 Mainz, Germany; markus.schepers@uni-mainz.de

**Keywords:** transperineal ultrasound, anal incontinence, anal sphincter, exoanal imaging

## Abstract

Background/Objectives: Anal incontinence is linked to pelvic floor dysfunction. Diagnosis involves assessing both the function and structure of the anorectal unit. Although transperineal ultrasound has gained attention as a less invasive option, its effectiveness as a diagnostic tool for evaluating the relationship between structure and function is still debated. This study aimed to explore the relationship between quantitative measurements of anal sphincter and pelvic floor structures as well as the subjective symptoms and objective assessments of sphincter function regarding anal incontinence. Methods: 50 women with pelvic floor dysfunction were recruited for the study. The severity of anal incontinence was assessed using the CACP score. Ultrasound imaging was employed to measure anal sphincter area, while sphincter pressures were evaluated through manometry. The relationships between variables were analyzed using Pearson’s and Spearman’s correlation tests. Results: The mean anal sphincter area was 5.51 cm^2^ at rest and 4.06 cm^2^ during maximal contraction. Resting anal sphincter pressure had an average of 46.29 mmHg, and contraction pressure averaged 103.25 mmHg. No significant correlation was found between the anal sphincter area and pressure at rest (r = 0.018) or during contraction (r = −0.210). However, a moderate correlation was observed between the change in sphincter pressure and area during contraction (r = 0.312). The CACP score showed no significant correlation with the sphincter area at rest (r = −0.084) but was weakly correlated during contraction (r = −0.270). Conclusions: Conventional diagnostic tools for evaluating anal incontinence can be uncomfortable and are not always readily available. Perineal sonography presents a promising, less invasive alternative for dynamic assessment of the anal sphincter.

## 1. Introduction

Anal continence is the ability to voluntarily control bowel movements appropriately in both time and place. In contrast, anal incontinence (AI), as defined by the International Continence Society (ICS), refers to the involuntary loss of fecal material or flatus [1]. Anal incontinence can be further categorized into fecal incontinence (FI), which refers to any involuntary loss of fecal material, and mirrors anal incontinence, except that it excludes flatus incontinence, which refers to any involuntary loss of gas (flatus). The uncontrolled loss of bowel contents is a distressing and disabling condition that leads to social isolation and has a major effect on quality of life. It also creates a significant financial burden and significantly contributes to both physical and psychological morbidity [2,3]. Estimating the severity of anal incontinence within the adult community has been difficult because people are often unwilling to share their incontinence symptoms. Prevalence rates vary depending on the definition used. In a national survey, approximately 8.3% of non-institutionalized adults aged 20 and older reported experiencing fecal incontinence [4]. A systematic review of fecal incontinence prevalence estimates found a range from 1.4% to 19.5%. Prevalence can increase with age, particularly in specific care settings, such as nursing homes, where it may reach up to 47% [2,5].

In clinical practice, most FI patients are women, likely due to pelvic floor anatomy and damage associated with obstetric trauma [6,7]. Fecal incontinence is considered a component of pelvic floor dysfunction, a condition affecting 25% or more of women, which additionally includes urinary incontinence and pelvic organ prolapse (POP) [8,9]. Since adequate anal continence is maintained by the structural and functional integrity of the anorectal unit, including muscular components such as the anal sphincter and the levator ani muscle, the main focus of the diagnostic approach is the assessment of the morphology and the evaluation of the function of the anorectal unit [6,10]. The best established diagnostic tool for assessing the function of the anorectal unit is anorectal manometry (ARM), which provides a direct evaluation of anal sphincter pressure and rectoanal coordination during simulated defecation [11]. However, interpreting ARM findings can be challenging due to the wide variability and overlap of manometric measurements in both health and disease [12]. Regarding morphology, endoanal sonography, introduced in the early 1990s, has become an essential method for diagnosing anal incontinence by evaluating the morphology of the anal sphincter, replacing traditional electromyography and establishing itself as the ‘gold standard’ in this context [3,13]. Over the past 30 years, research has focused on these methods and the relationship between the function and morphology of the anal sphincter complex [14,15].

The anal sphincter complex has gained significant attention from various disciplines, including obstetricians, due to the substantial role of obstetric anal sphincter trauma in the etiology of anal incontinence [14]. In the mid-1990s, due to endoanal sonography’s restricted availability in gynecological practice, the diagnostic option of “exoanal” transperineal sonography was introduced [16]. The exoanal imaging via transperineal ultrasound might be more accessible across various disciplines and will likely be less invasive for patients. Additionally, exoanal 3D/4D imaging provides a dynamic evaluation of the interaction between the pelvic floor viscera and musculature, allowing for a thorough evaluation of the entire pelvic floor [17]. The advancements in three-dimensional (3D) and four-dimensional (4D) transperineal ultrasound systems have enabled the visualization of the whole sphincter through tomographic ultrasound imaging (TUI) [18]. Tomographic transperineal ultrasound appears highly reliable and may serve as an alternative to endoanal imaging [19].

Studies comparing endoanal and transperineal ultrasound have demonstrated good agreement in diagnosing sphincter issues [20,21]. However, these studies did not address the subjective symptoms of fecal incontinence. Subsequent research has predominantly focused on the continuity of the muscle layer and the function of the anal sphincter complex, resulting in conflicting findings [22,23,24]. The current scientific understanding of the relationship between pelvic floor and sphincter morphology—both in terms of continuity and sphincter quality—and function, measured by pressure values and clinically perceived function, remains controversial. Studies investigating transperineal ultrasound for fecal incontinence have largely focused on the continuity of the muscle layer and the function of the anal sphincter complex. Our study aims to investigate the extent to which quantitative measures of anal sphincter and pelvic floor morphology correlate with both subjective and objective aspects of sphincter function.

## 2. Materials and Methods

**Figure 1:**
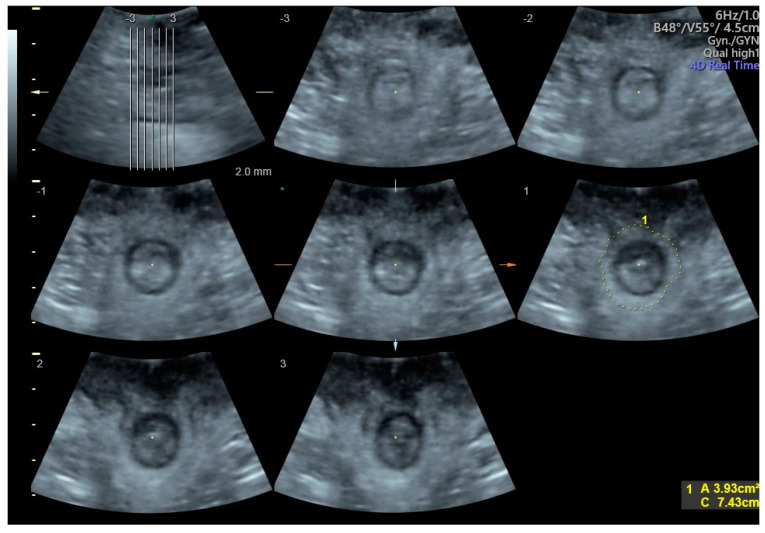
Visualization of the anal sphincter and measurement of its area using tomographic ultrasound imaging (TUI). The slices range from −3 to +3, and the green spot (*) marks the 0 point.

This study was designed as a pilot study. Ethical approval was received from the Ethics Committee of the State Medical Association of Rhineland-Palatinate (No: 2020-15278). Before recruitment, the study was registered in the German Clinical Trials Register (DRKS). Fifty non-pregnant women, aged 18 years and older, presenting with symptoms of pelvic floor dysfunction and referred to our urogynecological department between August 2021 and August 2022, were invited to participate in this research study. All participants provided written informed consent and were assessed for eligibility according to predefined inclusion and exclusion criteria. The exclusion criteria included: existing enterostomy (following bowel resection with neurolysis), current treatment with sacral neuromodulation due to insufficient contractility, presence of an artificial anal sphincter, and history of sphincteroplasty for a severe perineal tear. Each patient was routinely asked to complete the standard medical history form, which gathered information on the patient’s symptoms, which led to the consultation, and demographic data (e.g., age, parity, menopausal status, weight, height, medical history, and previous surgeries). The symptom severity of anal incontinence was assessed using the CACP (German Society for Coloproctology) score. This questionnaire, developed by the German Society for Coloproctology, covers stool frequency, consistency, and sensation during defecation, as well as episodes of incontinence and medication used for stool regulation [25]. A routine urogynecological examination was conducted, which included an assessment of pelvic organ prolapse, as well as an evaluation of pelvic floor muscle contraction using the modified Oxford scale [26]. Subsequent follow-up examinations included transperineal ultrasound with targeted sphincter sonography and anal manometry. The established measurement technique, as previously outlined by H.P. Dietz, was employed for the ultrasound assessment [17]. The hiatus urogenitalis (GH) area was measured at rest, during the Valsalva maneuver, and during the maximal pelvic floor contraction. The anal sphincter was visualized using tomographic ultrasound imaging (TUI). The number of slices and the spacing between them were adjusted based on the length of the sphincter, typically involving 6–7 slices with 2 mm spacing, as described in the literature [18]. In this study, the area of the anal sphincter was defined as the region outlined by tracing the outer hyperechoic boundary of the external anal sphincter. Measurements were taken from the imaging plane, where the sphincter complex was fully visualized (Figure 1). Following the transperineal ultrasound, anorectal manometry was used to assess resting and contraction pressures. All examinations were conducted using the Voluson™ P8 ultrasound device by GE Ultrasound Korea, Ltd. (Seongnam-Si, Republic of Korea). A convex transducer, the RAB2-6-RS abdominal transducer from GE Ultrasound Korea, Ltd., was utilized for perineal sonography. Anorectal manometry was performed with an SPM-2000 device by M&B Biomedizintechnik, Traunstein, Germany, and pressure measurements were conducted using a balloon pressure probe. No bowel preparation was given. Direct measurements and dynamic interactions were analyzed to assess the relationship between variables. Specifically, changes in the urogenital hiatus (in %) between resting, during Valsalva maneuver, and at maximal contraction, as well as changes in the area of the anal sphincter (in %) between resting and maximal contraction, and variations in anal sphincter pressure (in %) between resting and maximal contraction, were examined. The dynamic was referred to as the “Δ-value” in the analyses. Scatter plots were utilized to visually investigate the associations between various variables. Metric data were analyzed using both Pearson’s and Spearman’s correlation coefficients. A *p*-value of less than 0.05 was considered statistically significant. All statistical analyses were conducted using the IBM SPSS Statistics Version 25.

## 3. Results

### 3.1. Study Population and Demographics

The study cohort consisted of 50 patients, with a mean age of 59 years (range: 35–81) and a standard deviation of ±11 years. Most study participants (*n* = 36; 76%) were postmenopausal, while 24% (*n* = 12) were premenopausal. The mean body mass index (BMI) was 26.84 kg/m^2^ (range: 17.5–38.57), with a standard deviation of ±5.22 kg/m^2^. Among the participants, 40% had an average weight, 26% were overweight, and 33% were classified as obese. The mean parity was 2.06 (SD ±0.843). Eighty-eight percent (*n* = 44) of patients had at least one vaginal delivery. Five patients (10%) had a cesarean section in addition to vaginal deliveries, and one patient (2%) had only cesarean deliveries. One patient was nulliparous. Thirty-six percent (*n* = 18) of the patients showed no evidence of pelvic organ prolapse. As per POP-Q, the most prevalent finding was a prolapse in the anterior compartment (*n* = 23), which was primarily mild to moderate severity. According to the Oxford scale, the participants exhibited a mean pelvic floor contractility of three, with a standard deviation of 0.937. The mean CACP score was 14.36, with a standard deviation of 2.136. Most patients (82%, *n* = 41) obtained scores between 14 and 16 points, while three (6%) scored below 10, denoting diminished continence function.

### 3.2. Transperineal Ultrasound

**Figure 2:**
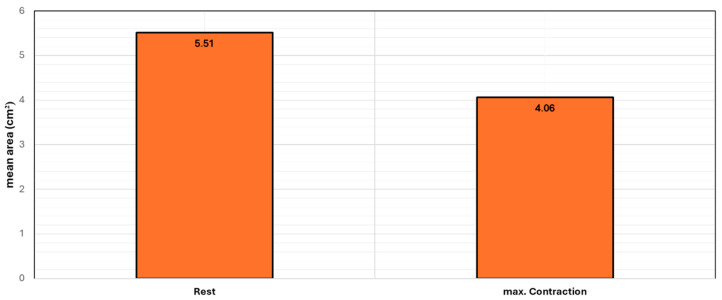
Bar chart diagram showing the mean values of the anal sphincter area (cm^2^) at rest and during maximal contraction.

In this study, the mean area of the urogenital hiatus was found to be 19.63 cm^2^ at rest (SD ±4.95 cm^2^), 16.82 cm^2^ during maximal contraction (SD ±4.66 cm^2^), and 26.58 cm^2^ during the Valsalva maneuver (SD ±7.44 cm^2^). The range of values for the urogenital hiatus area was observed to be at rest (12.46–36.11 cm^2^), during contraction (8.51–29.61 cm^2^), and during the Valsalva maneuver (15.20–58.36 cm^2^). According to the definition in the established literature [27], 48% (*n* = 24) of the patients exhibited a hiatus area within the normal range, whereas 26% (*n* = 13) displayed mild dilation, 16% (*n* = 8) had moderate dilation, 8% (*n* = 4) showed marked dilation, and only 2% (*n* = 1) had severe dilation under Valsalva. Furthermore, the mean area of the anal sphincter was measured to be 5.51 cm^2^ at rest (SD ±1.48 cm^2^) and 4.06 cm^2^ during maximal contraction (SD ±1.13 cm^2^). The range of values for the anal sphincter area was found to be (2.44–9.09 cm^2^) at rest and (1.86–7.15 cm^2^) during contraction. The study observed a reduction in the anal sphincter area during maximal contraction ranging from 2.48% to 66.9%, with a mean decrease of 25.22% and a standard deviation of 12.98% (Figure 2).

### 3.3. Anorectal Manometry

**Figure 3:**
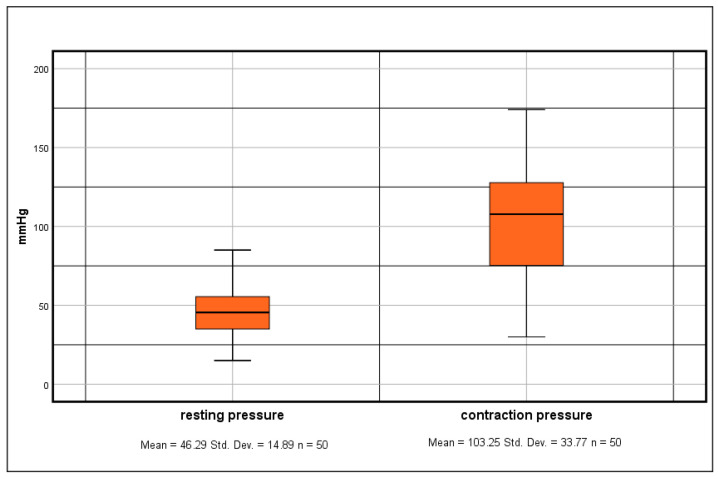
Box plot displaying the pressure values of the anal sphincter at rest and during contraction.

The resting anal sphincter pressure exhibited a range of 15 mmHg to 85 mmHg, while during maximal contraction, the pressure ranged from 30 mmHg to 174 mmHg. The average resting pressure was recorded at 46.29 mmHg (standard deviation ±14.89 mmHg), and the average contraction pressure at 103.25 mmHg (standard deviation ±33.77 mmHg), as illustrated in Figure 3. The elevation in anal sphincter pressure during maximal contraction varied from 20.67% to 597.62%, with a mean of 136.82% and a standard deviation of 100.02%.

### 3.4. Correlation Analysis

**Figure 4:**
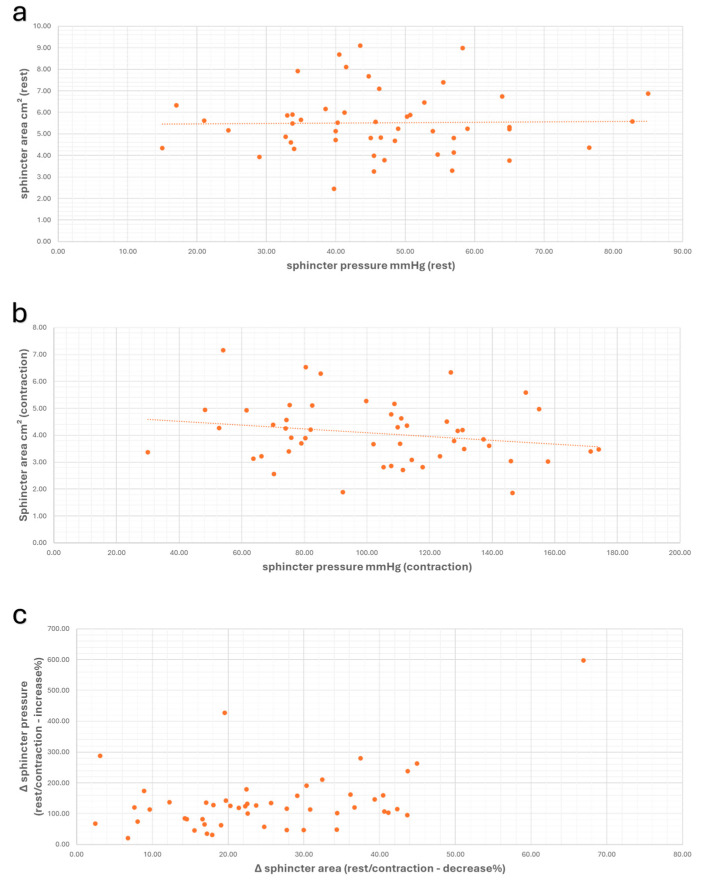
Scatter plots showing the values of anal sphincter pressure and anal sphincter area, along with the correlation between these values at rest (**a**) (r = 0.018, *p* = 0.899), during contraction (**b**) (r = −0.210, *p* = 0.144), and the correlation of Δ-values (**c**) (r_s_ = 0.312, *p* = 0.028).

In this study, the primary focus was on investigating the functional relationship between the area of the anal sphincter (in cm^2^) and sphincter pressure. In this context, a comparison between sonographically-measured sphincter area and manometrically-recorded pressure values at rest and during contraction was evaluated. These variables showed no significant correlation within the study population (at rest: r = 0.018, *p* = 0.899; during contraction: r = −0.210, *p* = 0.144). However, as would be reasonably expected, the increase in anal sphincter pressure (Δ sphincter pressure) moderately correlates with the decrease in sphincter area (Δ sphincter area) during maximal contraction (r_s_ = 0.312/*p* = 0.028). The sphincter pressure increased as the sphincter area decreased (Figure 4).

**Figure 5:**
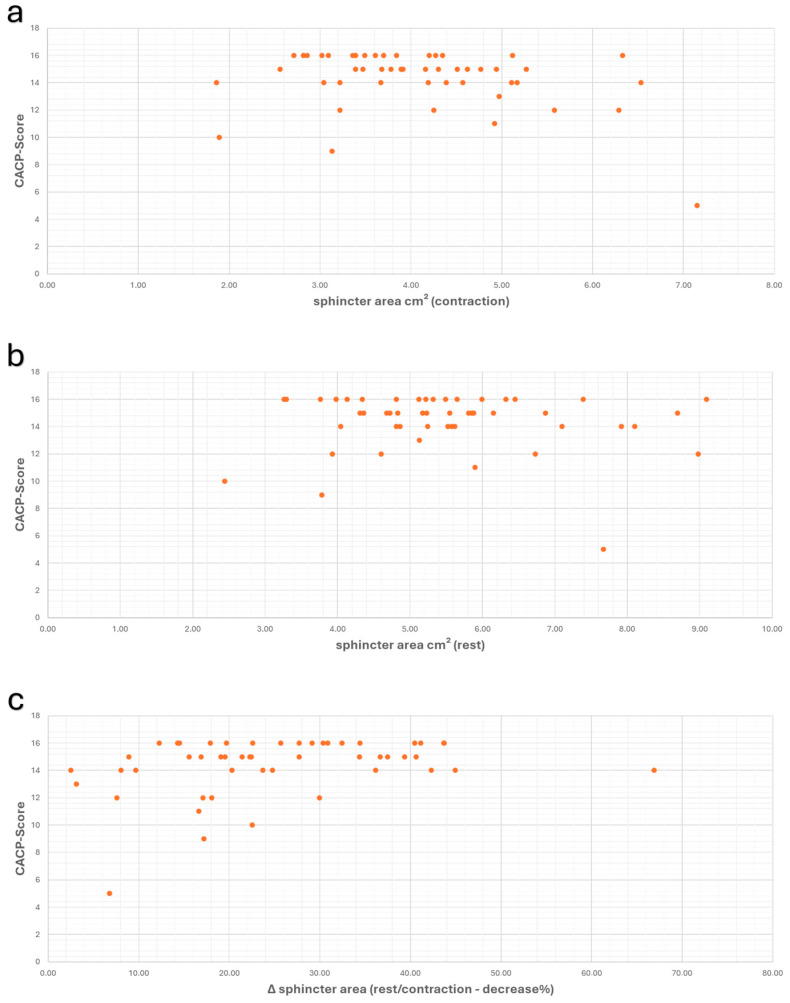
Scatter plots showing the values of anal sphincter area and CACPScore, along with the correlation between these values during contraction (**a**) (r_s_ = −0.270/*p* = 0.058), at rest (**b**) (r_s_ = −0.084/*p* = 0.561), and the correlation of Δ-values (**c**) (r_s_ = 0.315/*p* = 0.026).

A comparison of the CACP score with the sonographically-assessed sphincter areas at rest and during contractions revealed a weak relationship between these two variables during contraction but no significant correlation at rest (at rest: r_s_ = −0.084/*p* = 0.561; during contraction: r_s_ = −0.270/*p* = 0.058). The comparison of the Δ-values of sphincter area and CACP scores demonstrated a moderate positive correlation (r_s_ = 0.315/*p* = 0.026) (Figure 5).

The assessment revealed no statistically significant correlation between the subjective anal continence function, as evaluated by CACP score, and anal sphincter pressure, both at rest (r_s_ = 0.188, *p* = 0.192) and during contraction (r_s_ = 0.172, *p* = 0.234). Subjective continence in the study population was not associated with the measured anal sphincter pressure. Furthermore, the CACP score did not correlate with the increase in anal sphincter pressure either (Δ sphincter pressure) (r_s_ = 0.092/*p* = 0.524).

**Figure 6:**
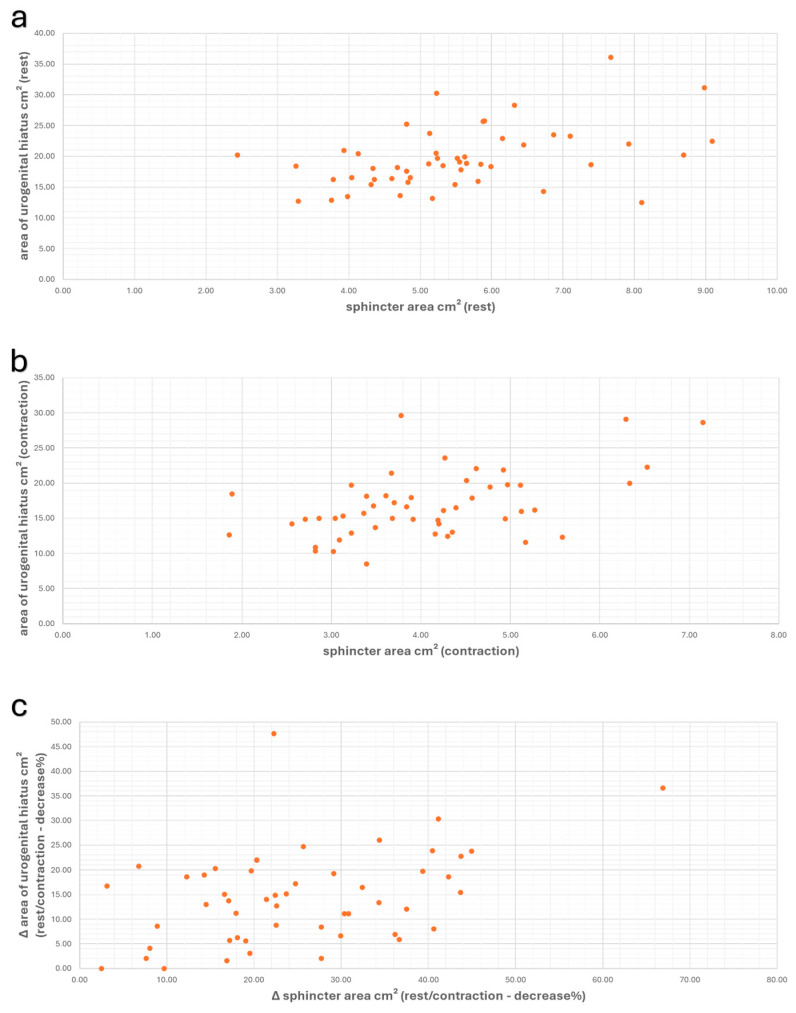
Scatter plots showing the values of the area of GH and anal sphincter area, along with the correlation between these values at rest (**a**) (r_s_ = 0.450/*p* = 0.001), during contraction (**b**) (r_s_ = 0.443/*p* = 0.001), and the correlation of Δ-values (**c**) (r_s_ = 0.363/*p* = 0.009).

The comparison of the values for the anal sphincter area and the area of the urogenital hiatus, both at rest and especially during contraction, showed a significant moderate correlation (at rest: r_s_ = 0.450/*p* = 0.001, and during contraction: r_s_ = 0.443/*p* = 0.001). Accordingly, a statistically significant positive correlation was also demonstrated when comparing the Δ-values of these parameters (r_s_ = 0.363/*p* = 0.009) (Figure 6).

**Figure 7:**
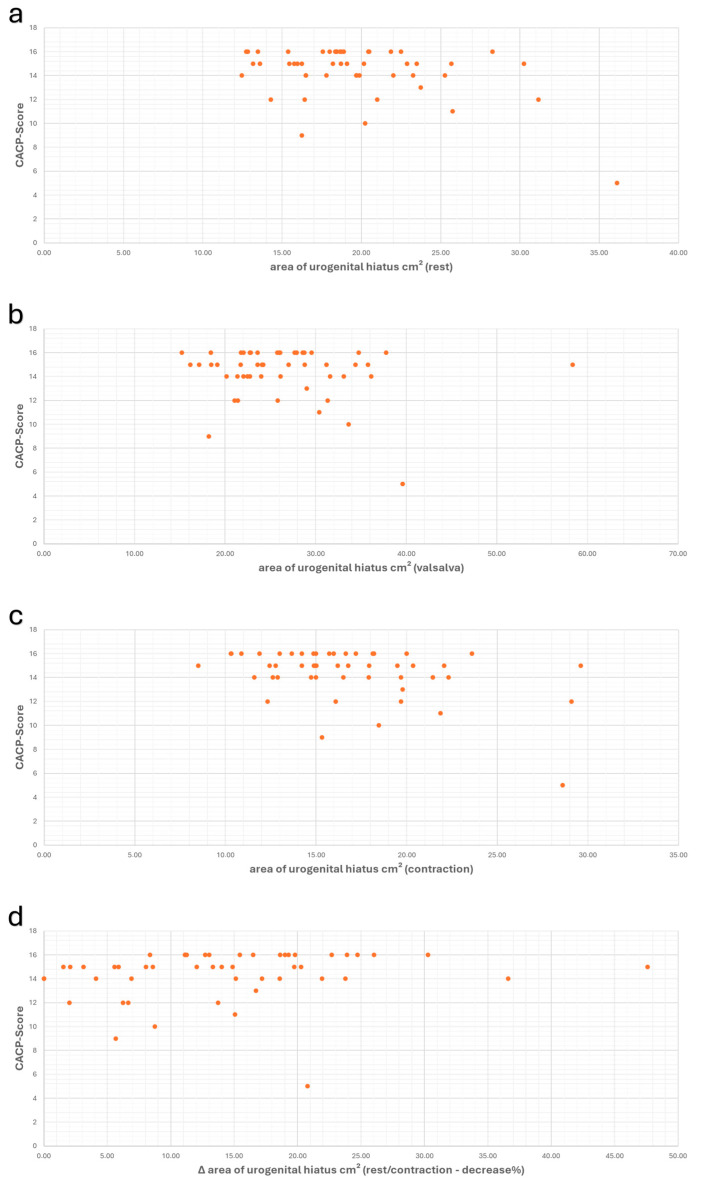
Scatter plots showing the values of the area of GH and CACP-Score, along with the correlation between these values at rest (**a**) (r_s_ = −0.212/*p* = 0.140), during Valsalva (**b**) (r_s_ = −0.056/*p* = 0.698), during contraction (**c**) (r_s_ = −0.289/*p* = 0.042), and the correlation of Δ-values (**d**) (r_s_ = 0.291/*p* = 0.041).

There was no significant correlation between the CACP score and the area of the urogenital hiatus at rest or during the Valsalva maneuver (at rest: r_s_ = −0.212/*p* = 0.140, and during Valsalva: r_s_ = −0.056/*p* = 0.698). The hiatus area during contraction and the change in hiatus area (Δ hiatus area) showed a weak correlation with the CACP score (during contraction: r_s_ = −0.289/*p* = 0.042, and Δ-values: r_s_ = 0.291/*p* = 0.041) (Figure 7).

The comparison of anal sphincter pressure values at rest and during contraction with the area of the urogenital hiatus, both at rest and during contraction, showed no correlation at rest (r_s_ = −0.04/*p* = 0.784) and a significant correlation during contraction (r_s_ = −0.341/*p* = 0.015). A weak correlation was observed when comparing the Δ-values of these parameters, but it did not reach significance (r_s_ = 0.253/*p* = 0.076).

The comparison of pelvic floor contractility values assessed using the Oxford scale with the sonographic area of the urogenital hiatus, both at rest and, particularly, during contraction, showed a significant moderate correlation (at rest: r_s_ = −0.364/*p* = 0.009, and during contraction: r_s_ = −0.531/*p* < 0.001). Accordingly, a statistically significant positive correlation was also demonstrated when comparing the Oxford scale values with the Δ-values of these parameters (r_s_ = 0.406/*p* = 0.003). Pelvic floor contractility correlated with the area of the urogenital hiatus both at rest and during contraction. Pelvic floor contractility was assessed using the Oxford scale, and the measure of sphincter area reduction (Δ sphincter area) showed a weak but insignificant correlation (r_s_ = 0.263/*p* = 0.065).

The comparison of pelvic floor contractility values assessed using the Oxford scale with manometric sphincter pressure during contraction showed a significant positive correlation (r_s_ = 0.536/*p* < 0.001). The Oxford scale also correlated positively with Δ sphincter pressure (r_s_ = 0.390/*p* = 0.005). In contrast, no correlation was observed between resting pressure and pelvic floor contractility (r_s_ = 0.136/*p* = 0.345). The comparison between pelvic floor contractility, assessed using the Oxford scale, and subjective continence function, measured by the CACP score, showed, as expected, no significant correlation (r_s_ = 0.140/*p* = 0.331) (Table 1).

In the context of this study, subjective continence showed no significant correlation with parity, the number of spontaneous births, or BMI. However, subjective continence statistically significantly decreased with increasing age.

## 4. Discussion

This study primarily focused on evaluating the 2D and 3D sonographic characteristics of the anal sphincter through transperineal ultrasound, aiming to assess both objective and subjective functions. Initially, the area of the anal sphincter was compared with manometric pressure values and clinical continence function concerning anal incontinence. Additionally, a secondary analysis was performed to assess the correlation of other sonographic measurements of the pelvic floor with anal incontinence.

The area measurement of the anal sphincter was performed using transperineal sonography. To our knowledge, this type of dynamic measurement for correlation analyses at rest and during maximal contraction has been conducted for the first time in our study. Similarly, Huang et al. investigated data from 55 nulliparous women in a cohort study, where the sonographic measurements of the anal sphincter were also obtained using exoanal sonography [28]. However, the authors differentiated individual components of the anal sphincter in their study: the anal mucosa, the external anal sphincter muscle, and the internal anal sphincter muscle. The summed mean area of the various components of the anal sphincter in this study at rest was (0.92 + 1.59 + 3.20) 5.71 cm^2^. This is comparable to our measurement data. However, a measurement of the anal sphincter area during contraction was not conducted in this study. The measurement parameters of the anal sphincter described in the literature, obtained through endoanal sonography, are, on the other hand, not comparable to our data, as the anal sphincter is distended when the ultrasound probe is inserted [29]. Although the endoanal and exoanal measurement methods have different normative values, each technique appears to be applicable for visualizing and measuring the dimensions of the anal sphincter.

Patients suffering from anal incontinence [6] generally show lower manometric pressure values. However, no correlation between the manometric pressure values of the anal sphincter and subjective clinical continence has been demonstrated in the literature [30]. In our study, the comparison of the CACP scores and manometric pressure values showed no significant correlation regarding clinical continence function for resting pressure, squeeze pressure, or pressure increase (Δ sphincter pressure) (r_s_ = 0.188, *p* = 0.192; r_s_ = 0.172, *p* = 0.234; r_s_ = 0.092, *p* = 0.524). Our results align with the existing literature. However, it should be critically noted that our small cohort included only a few patients with anal incontinence. Nevertheless, the reproducibility and validity of sphincter manometry in anal incontinence remain unclear.

We investigated the correlation between the sonographically-measured anal sphincter area and anal sphincter pressure as the primary focus of this study. The comparison of these values showed no significant correlation between the absolute area of the anal sphincter and the manometric pressure values of the anal sphincter. However, the dynamic measurement of the sphincter area, that is, the reduction in the area of the anal sphincter compared to the resting area, showed a significant correlation with the corresponding manometric pressure values (r_s_ = 0.312/*p* = 0.028) as well as with clinical continence function (r_s_ = 0.315/*p* = 0.026). In our study, we observed an average reduction in the volume of the anal sphincter area by 25.22%. With the decrease in sphincter area, an increase in sphincter pressure is observed. However, in the absence of multivariate analyses regarding the correlation between sphincter dimensions and continence function, substantial evidence about the correlation between the two needs to be provided. In the literature, relatively heterogeneous results can be found on this topic. Sultan et al. conducted a prospective study involving 93 nulliparous women and 21 healthy men [14]. The authors performed measurements of the anal sphincter using endoanal ultrasound and found no correlation between sphincter thickness and anal sphincter pressure. Schäfer et al. examined the correlation between the manometric pressure values of the anal sphincter and sphincter thickness [31]. This study group consisted of patients with continence issues. In contrast to the study by Sultan et al., the authors demonstrated a significant correlation between squeeze pressure and the thickness of the external anal sphincter. However, the correlation coefficient in this study remained relatively weak (r = 0.27), allowing for various interpretations. Law et al. showed in a smaller study a significant correlation between the thickness of the internal anal sphincter and the manometric resting pressure of the anal sphincter [32]. However, this relationship was no longer present when the sphincter exhibited a disruption indicative of discontinuity. These studies were conducted in different study populations and with varying ultrasound probes. Titi et al. studied patients with anal incontinence [33]. In this study, a significant correlation was found between the maximum thickness of the external anal sphincter and the squeeze pressure of the anal sphincter. This correlation was not observed between the internal anal sphincter and the resting pressure of the anal sphincter. In contrast, the correlation between the mean thickness of the external anal sphincter and the squeeze pressure was borderline. Patients with anal sphincter injuries were not excluded from this study, resulting in a degree of heterogeneity. West et al. conducted a randomized study using 3D ultrasound to investigate the correlation between anal sphincter volume and sphincter pressure. They found no correlation between the muscle volume of the anal sphincter and clinical anal incontinence [34]. Fowler et al. and Titi et al. also explored the relationship between the length/thickness of the anal sphincter and clinical continence function [33,35]. The authors were unable to demonstrate a significant correlation between clinical continence function and the dimensions of anal sphincter. In our study, no correlation was found between clinical continence function, measured using the CACP score, and anal sphincter area (resting r_s_ = −0.084/*p* = 0.561 and during contraction r_s_ = −0.270/*p* = 0.058). However, there were too few patients with incontinence in our study cohort, making any connection questionable. Nevertheless, our results align with the existing literature. The individual measurements of the anal sphincter area at rest and during contraction showed no correlation with manometric measurements and clinical continence function.

A healthy pelvic floor is essential for maintaining the proper position of the pelvic organs and ensuring continence. Among the various muscles in the pelvic floor, the levator ani stands out as the most important, playing a key role in its overall function. Effective pelvic floor training can reduce symptoms of anal incontinence. This also suggests a potential role of the contractility of the levator ani muscle in maintaining anal continence [36]. The existing scientific literature provides no clear conclusions on this matter, as the studied populations, diagnostic methods, and follow-up durations vary significantly. In our study, pelvic floor contractility was assessed both clinically and sonographically. The degree of reduction in the hiatus area during maximum contraction indicated pelvic floor contractility. Clinically, the Oxford scale was used to assess the contractility. The literature on pelvic floor contractility is controversial. Objective measurements from perineal sonography indicate a correlation, but this cannot be confirmed through subjective clinical assessment. Fernandez-Fraga et al. investigated the relationship between pelvic floor contractility and anal incontinence in a prospective study [37]. This study objectively measured pelvic floor contractility using a perineal dynamometer. The authors found a significant correlation between anal incontinence and pelvic floor contractility. However, this study did not account for injuries to the anal sphincter and the levator ani muscle. Oversand et al. conducted a retrospective study involving 726 patients and, in contrast to Fernandez-Fraga et al., found no significant correlation between pelvic floor contractility and anal incontinence [38]. This study clinically measured contractility using the Oxford scale and sonographic parameters obtained via perineal sonography. A weak negative correlation was observed only between the reduction in the diameter of the pelvic inlet during contraction and anal incontinence. Furthermore, this retrospective study did not include assessments regarding the continuity or injury of the anal sphincter and the levator ani muscle. Our analyses showed a significant correlation between sonographically-measured contractility (Δ area of GH) and the continence score (CACP) (r_s_ = 0.291, *p* = 0.041), which addresses a significant correlation between anal continence function and pelvic contractility, measured with an objective method. The higher the contractility, the lower the incontinence score. Our study indicates that there is a weak correlation between anal continence performance and the decrease in the hiatus area in perineal sonography. Without multivariate analysis, these results are initially consistent with the literature [37,38]. However, this correlation was not evident in the correlation analysis between pelvic floor contractility, when measured using the Oxford scale, and CACP scores (r_s_ = 0.140, *p* = 0.331). Although the Oxford scale is the most used method for assessing pelvic floor contractility, it notably depends on the examiner [39]. Furthermore, the literature interestingly shows that nearly half of women are unable to adequately contract their pelvic floor. This can also explain our controversial results and those in the literature regarding contractility. However, the strength of the evidence remains weak due to the lack of prospective randomized studies.

The proper contractility of the pelvic floor, specifically the levator ani muscle, is also influenced by its structural integrity, among several other factors [40]. A rupture of the levator ani muscle from its insertion point leads to an expansion of the genital hiatus, which significantly increases the risk of pelvic organ herniation, resulting in genital descent [41]. Measuring the hiatus area at rest and during the Valsalva maneuver using perineal sonography allows for assessing pelvic floor dysfunction risk even in patients with an intact levator ani muscle [42]. A large area of the urogenital hiatus and injuries to the levator ani muscle correlate, according to the literature, only with descent in the anterior vaginal compartment, manifesting as a cystocele, as well as in the central compartment, manifesting as prolapse of the uterus or vaginal stump [41,43]. However, no significant correlation can be demonstrated between the area of the urogenital hiatus and anal continence function [40]. In the present study, pelvic floor defects were not assessed clinically. Although patients with a history of sphincteroplasty due to severe perineal tears were excluded from the study, additional pelvic floor defects were not evaluated. However, the gap in examining the levator ani’s integrity may be overlooked in the current study protocol. The literature data shows that a levator ani injury is not an independent risk factor for anal incontinence in the case of an intact anal sphincter [44,45,46]. In our study, the comparison of the CACP scores and the sonographic area of the genital hiatus at rest and during the Valsalva maneuver revealed no significant correlation (r_s_ = −0.212, *p* = 0.140; r_s_ = −0.056, *p* = 0.698). These findings are consistent with the literature. Furthermore, the significant correlation between the measurements of the urogenital hiatus area and anal sphincter area (r_s_ = 0.363/*p* = 0.009) reveals important information about the interplay of pelvic floor components concerning anal incontinence.

### Limitations of the Study

This study included patients from the urogynecological outpatient clinic, where it can be assumed that each patient experienced some limitations in pelvic floor function. The patient cohort exhibited a diverse age distribution, and the rate of anal incontinence among them corresponded to that of the general population. The objective was to assess the significance of perineal sonography in evaluating the anal sphincter. The heterogeneity regarding anal incontinence and other pelvic floor dysfunctions complicates the generation of precise results. Pelvic floor contractility was assessed using the Oxford scale; however, this examiner-dependent method limits the significance of the results. The study measured Δ sphincter pressure to compare related values, as individual contractility is better expressed this way. However, this Δ does not reflect the specific function of any sphincter component. The internal anal sphincter generates the most resting pressure, while the external anal sphincter provides squeeze pressure. The Δ sphincter pressure indicates the additional pressure exerted by the external anal sphincter but cannot be attributed to any specific function of the involved components.

A performed post hoc power analysis using G*Power version 3.1.9.7 showed that given a population effect size of r = 0.30, computed from the correlation between sphincter morphology and function, a sample size of 50, an α level of 0.05, and a two-tailed test, has a low calculated power (0.57). Therefore, we had inadequate power to confidently detect the correlation between sphincter morphology and function for such a small effect size. A larger sample size would be necessary to achieve a power of 0.80, allowing for more reliable detection of correlations of similar or smaller magnitudes. Therefore, the results must be cautiously evaluated and interpreted carefully.

The incidence of incontinence is expected to rise in the coming years. A population-based projection study indicates that demographic changes may lead to a 60% increase in pelvic floor dysfunctions, including anal incontinence, in the first half of the century [47]. Therefore, improving diagnostic and therapeutic methods is essential. Increased awareness of these symptoms, particularly among women, and the availability of accessible examination methods such as transperineal sonography can facilitate patient access. Measuring the anal sphincter area provides valuable insights into the function of the continence apparatus and correlates with established diagnostic methods. A thorough assessment of the pelvic floor using transperineal ultrasound, including the anal sphincter, is a robust method for diagnosing pelvic floor function. Adding dynamic measurements of structure can further improve the evaluation. However, further extensive studies are needed to address these issues and strengthen the evidence in this field.

## Figures and Tables

**Table 1:** Association of pelvic contractility and anal continence with dynamic variables.

	Δ Sphincter Area	Δ GH Area	Δ Sphincter Pressure	Oxford-Scale	CACP-Score
Oxford-Scale	ns	r_s_ = 0.406 *p* = 0.003	r_s_ = 0.390 *p* = 0.005	-	ns
CACP-Score	r_s_ = 0.315 *p* = 0.026	r_s_ = 0.291 *p* = 0.041	ns	ns	-

## Data Availability

The data are not publicly available due to ethical restrictions. However, they are available from the corresponding author upon reasonable request and with permission from the relevant institution.
